# Impact of Multifaceted Interventions Including Waterless Patient Care on Endemic Occurrence of *Serratia marcescens* in an Intensive Care Unit

**DOI:** 10.3390/pathogens14040363

**Published:** 2025-04-08

**Authors:** Romain Martischang, Gaud Catho, Abdessalam Cherkaoui, Filippo Boroli, Niccolo Buetti, Jerome Pugin, Stephan Harbarth

**Affiliations:** 1 Infection Control Program, WHO Collaborating Center for Patient Safety, Geneva University Hospitals, Rue Gabrielle-Perret-Gentil, 4, CH-1205 Geneva, Switzerland; 2 Infectious Diseases Division, Central Institute, Valais Hospital, CH-1950 Sion, Switzerland; 3 Bacteriology Laboratory, Geneva University Hospitals, CH-1211 Geneva, Switzerland; 4 Division of Intensive Care Medicine, Geneva University Hospitals, CH-1205 Geneva, Switzerland

**Keywords:** *Serratia marcescens*, sink, waterless, outbreak, aquatic reservoir, critical care

## Abstract

*Serratia marcescens* acquisition is a common problem in intensive care units (ICUs). Following an initial outbreak in 2017 with ongoing endemicity, this study aimed to analyze the impact of behavioral interventions and sink removals on *S. marcescens* incidence in a tertiary-care ICU. We conducted a quasi-experimental, interventional study including patients with a positive screening or clinical culture for *S. marcescens*, from 48 h (D2) after ICU admission to 14 days after ICU discharge. A sub-analysis considered patients positive for *S. marcescens* from ICU admission (D0) to 14 days after ICU discharge. Multivariate Poisson regression analyses were performed. Between January 2014 and December 2022, 167 cases of *S. marcescens* infection or colonization were identified (respiratory samples, 71%). Despite the presence of an aquatic reservoir, we found that neither behavioral nor architectural interventions (sink removal) reduced significantly *S. marcescens* incidence, yielding incidence ratios of 1.02 [95%CI 0.33–3.11] and 4.25 [95%CI 0.59–30.56], respectively. However, an association was observed with administration of selective oral decontamination (SOD) in the sub-cohort (OR 1.01; 95%CI 1.00–1.03). Behavioral change interventions and transition to a waterless ICU did not control endemic, polyclonal *S. marcescens* occurrence. The selective pressure exercised by SOD may have reduced the effectiveness of waterless care.

## 1. Introduction

*Serratia marcescens* is an aerobic Gram-negative pathogen, which may colonize digestive and respiratory tracts and cause various infectious syndromes. Its environmental reservoirs, ranging from medical equipment (e.g., respirators, nebulizers) to hospital sinks [1,2,3] and its persistence for up to 24 months [4], contribute to its notoriety for causing nosocomial outbreaks that are difficult to eradicate. Infection control efforts traditionally focus on measures such as improving hand hygiene [5], staffing workload [6,7], catheter bundles, and water-related practices, rather than pathogen-specific precautions. However, controlling the risk related to aquatic reservoirs such as sinks in intensive care units (ICUs) remains a logistical challenge (e.g., sink disinfection, trap replacements) [8].

The adult ICU at Geneva University Hospitals (HUG) experienced the occurrence of endemic, multi-susceptible *S. marcescens*, revealed during the investigation of an outbreak between July and October 2017, affecting 16 critically ill patients, including 9 infections with 2 cases of bloodstream infections. A thorough epidemiological, microbiological, and genomic investigation linked this outbreak to sinks, triggering multiple behavioral measures to enhance hand hygiene and sink-related practices [9]. A subsequent outbreak of VIM-producing *P. aeruginosa* from April 2018 to September 2020 was successfully controlled following architectural and procedure changes [10], notably the removal of sinks, to eliminate water reservoirs and allow water-free care. This report aims to assess the impact of combined behavioral and architectural interventions on controlling endemic *S. marcescens* in this tertiary care ICU.

## 2. Materials and Methods

### 2.1. Study Design

We conducted a quasi-experimental study spanning from 1 January 2014 to 31 December 2022 (intervention period: March 2018–December 2022) to assess the sequential impact of behavioral and architectural interventions.

### 2.2. Setting and Population

The adult mixed surgical–medical ICU at HUG is divided into two sectors (A and B) on the same floor, accommodating 30 beds serving approximately 2500 patients yearly, with a median length of stay of 4 days. During the COVID pandemic waves the full capacity went up to 48 beds [11]. Sector A features 3–4 bed rooms, each with a medication preparation area with a sink, and a compact utility room equipped with a sink and a washer–decontaminator. Sector B has single-bed rooms, each furnished with a sink and a washer–decontaminator.

### 2.3. General Infection Control and Surveillance Measures

Monthly hand hygiene compliance among healthcare workers was monitored using WHO protocols. Systemic antibiotic consumption was measured in defined daily doses (DDD) and included consumption of antibiotics at risk of selecting *S. marcescens* (Supplementary file S1 in Supplementary Materials), calculated per 1000 patient days. Beginning in March 2015, as part of a horizontal measure to prevent ventilator-associated pneumonia (VAP), patients on ventilation for more than 48 h underwent thrice-daily selective oropharyngeal decontamination (SOD) with colistin, tobramycin, and amphotericin B [12]. Ventilation days were also recorded monthly, as described previously [12]. The onset of the COVID-19 pandemic in March 2020 altered screening and infection control practices, including a temporary adoption of universal gloving that was stopped in 2021.

### 2.4. Interventions

To control the initial *S. marcescens* outbreak, we conducted audits to assess infection control practices among healthcare workers in August 2017, water-related activities in November 2018, and activities related to the maintenance of ventilatory circuits in July 2021. Behavioral interventions, executed from February to March 2018, included training sessions and establishing procedures to enhance standard precautions, particularly for water-related tasks like patient toileting and waste management. From March 2020, contaminated and non-contaminated areas were segregated, with the creation of dedicated storage spaces more than one meter away from sinks.

In addition to these measures, architectural interventions were conducted in September 2020, to eliminate the aquatic reservoir by implementing water-free patient care, removing all sinks from patient rooms, and most of the sinks in medication preparation areas. Only the sinks in the compact utility room designated for waste disposal and two sinks in the preparation rooms were retained. Concurrently, water-free patient care-related activities were introduced [10]. A complete list of conducted interventions and preventive measures, including hand hygiene and ventilation circuit management, can be found in Table S1.

### 2.5. Microbiological Methods and Surveillance

To address the *S. marcescens* outbreak detected in August 2017, we conducted respiratory and rectal screening of ICU patients in September 2017. Additionally, we performed two environmental sampling surveys in August 2017 and 2018, including respectively 56 and 30 samples from various sources including surfaces, sinks, U-bends, and beds before and after disinfection (Supplementary file S2 for further methods). All environmental samples were cultured. Colonies presumptive for *S. marcescens* were identified by MALDI-TOF/MS. We also conducted genomic analyses of the 2017 outbreak strains, along with strains randomly sampled from 2015 to 2018 (*n* = 69) and 2 environmental isolates to help understand the genetic relatedness of circulating *S. marcescens* strains (Supplementary file S3).

### 2.6. Interventional Cohort Study with Interrupted-Time Series Analysis

Our main cohort encompassed ICU patients from January 2014 to December 2022 who had a screening or clinical culture positive for *S. marcescens* more than 48 h (D2) after ICU admission and up to 14 days post-discharge. Two sensitivity analyses were also performed: SCohort 1 included patients with any *S. marcescens*-positive result from time zero (D0) of ICU admission to 14 days after ICU discharge, and SCohort 2 included patients with a first negative screening or clinical sample (urine or respiratory sample) during ICU stay, and with a subsequent screening or clinical sample positive for *S. marcescens* more than 72 h after ICU admission. Only the first isolate per patient was considered. Consecutive ICU stays shorter than 30 days were merged to account for repeated admissions. We collected patient-related variables (SAPS II, length of stay in days, ventilation days), infection control metrics (hand hygiene adherence, antibiotic pressure, number of daily SOD prescriptions, as defined above), and healthcare workload as the project research of nursing score (PRN).

Continuous variables were reported as mean (±SD) or median (IQR), based on their distribution. Monthly time-varying exposures were compared using the chi-square test, *t*-test or Wilcoxon test. We performed interrupted time-series analyses to assess changes in *S. marcescens* trends and levels following behavioral and architectural interventions [13]. Study periods were a 50-month baseline (1 January 2014–28 February 2018), a 31-month period with the behavioral intervention (1 March 2018–30 September 2020), and a 27-month period with the architectural intervention (1 October 2020–31 December 2022).

We defined the incidence of new *S. marcescens* cases as the main outcome of interest, offset by monthly ICU patient days. Univariate and multivariate Poisson regression models were applied with predetermined time-varying covariates (SAPS II including the age component, PRN, length of stay, hand hygiene adherence, ventilation days, antibiotic pressure, and number of SOD prescriptions). Temporal effects were accounted for using a study phase × study month interaction term, and a seasonal factor using calendar months. All possible models (Supplementary file S4), were compared using the Akaike information criterion. Model robustness was assessed via autocorrelation, partial autocorrelation functions, and residual plots. Subgroup analyses for SCohort 1 and SCohort 2 employed quasi-Poisson models to address overdispersion and Fourier terms for seasonality. Statistical analyses were conducted using RStudio software (RStudio Team (2015)).

## 3. Results

**Figure 1:**
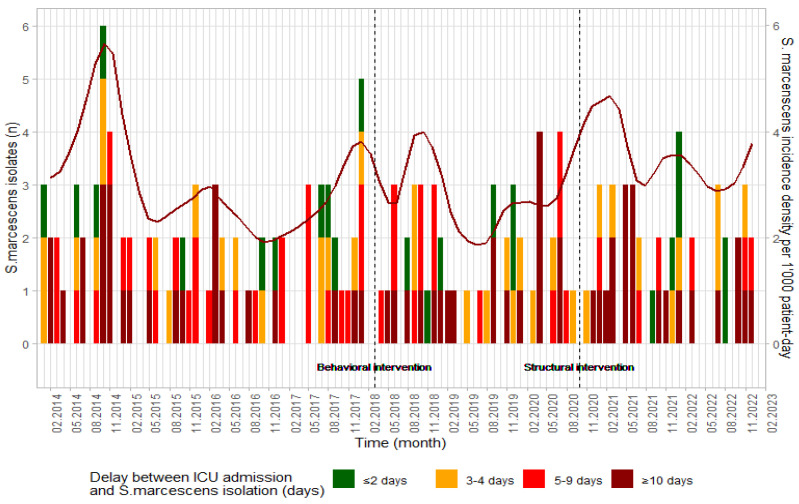
Epidemiologic curve of *Serratia marcescens* detected in the intensive care units at HUG, 2014 to 2022.

From January 2014 to December 2022, 167 patients were identified with either a colonization or an infection with a multi-susceptible *S. marcescens* strain, detected >48 h of ICU admission and up to 14 days after ICU discharge (Figure 1). Of these, 42 patients (25.2%) were identified only after ICU discharge.

### 3.1. Interrupted Time-Series Analysis

From January 2014 to December 2022, the breakdown across different periods was as follows: 79 patients (2.74 cases per 1000 patient days) were identified with *S. marcescens*-positive culture during the baseline period, 47 patients (1.69 per 1000 patient days) during the behavioral intervention period, and 41 patients (2.95 per 1000 patient days) during the architectural intervention period (Table 1). SCohort 1 encompassed 233 cases, and SCohort 2 encompassed 46 cases. Ventilation days, PRN and SAPS II scores, length of stay, antibiotic consumption, and SOD prescriptions were statistically higher during the architectural intervention period compared to the control and/or behavioral intervention periods (Table 1). Most *S. marcescens* isolates were identified in respiratory samples (70.7%, 118/167), followed by blood (13.2%, 22/167), and urine (7.8%, 13/167). Further details on the SCohorts can be found in Table S2.

From a total of 1023 evaluated multivariate models, the optimal model (AIC = 110.31) included the PRN score, hand hygiene compliance, antibiotic consumption, and frequency of SOD prescriptions. This model revealed that neither behavioral nor architectural interventions significantly reduced *S. marcescens* incidence, yielding incidence ratios of 1.02 [95%CI 0.33–3.11] and 4.25 [95%CI 0.59–30.56], respectively. PRN score (1.00 [95%CI 0.99–1.00]), hand hygiene compliance (1.01 [95%CI 0.97–1.05]), antibiotic consumption (0.99 [95%CI 0.97–1.00]), and the number of SOD prescriptions (1.01 [95%CI 0.98–1.03]) did not show statistical significance either (Table S3).

When considering patients enrolled in SCohort 1, including all cases of *S. marcescens* from D0, the best model included the periodical monthly variable, PRN score, hand hygiene compliance, antibiotic consumption, and the frequency of SOD prescriptions. The months of January and February, along with the number of SOD prescriptions, showed statistical significance, with incidence ratios of 2.36 [95%CI 1.01–5.48] (*p* = 0.046), 0.30 [95%CI 0.09–1.00] (*p* = 0.05), and 1.01 [95%CI 1.00–1.03] (*p* = 0.01), respectively (Table S4). When considering SCohort2, including only ICU-acquired *S. marcescens* cases in patients with negative baseline samples, the best model included the PRN score, hand hygiene compliance, ventilation days, antibiotic consumption, and SOD prescription frequency. However, this model did not reveal any statistically significant associations (Table S5), except a borderline significance of antibiotic consumption with an incidence ratio of 0.99 [95%CI 0.97-1.00] (*p* = 0.08).

### 3.2. Environmental Screening and Audits

Environmental screening detected two *S. marcescens*-positive ICU sinks. A first audit highlighted environmental issues with ICU sinks in sector A, notably their proximity to washer–disinfectors, contamination potential from nearby materials, and challenges related to hand hygiene and biological waste disposal. Similar concerns were noted in sector B, where sinks located in patient rooms were found to be inadequately sized and shallow. A second audit identified contamination risks associated with the maintenance of respirator equipment in both non-invasive and invasive ventilation procedures, with inconsistent management of filters, risks during extended use of respiratory circuits without disconnection, and during temporary disconnection.

### 3.3. Genomic Analysis 

Further core-genome MultiLocus Sequence Typing (cgMLST) analysis of strains collected between 2015 and 2018 across different HUG departments ruled out the presence of a monoclonal cluster explaining the ICU outbreak in 2017 (Figure S1).

## 4. Discussion

This study identified endemic spread of polyclonal *S. marcescens* in our ICU, interspersed with epidemic peak periods. Insights gleaned from epidemiological and molecular analyses contributed to two main conclusions: firstly, neither behavioral interventions nor the implementation of waterless patient care effectively curtailed the endemic occurrence of *S. marcescens*; the assumed aquatic reservoir as a source of *S. marcescens* acquisitions remains speculative and ambiguous since sink removal did not impact incidence of *S. marcescens*; secondly, SOD may be a risk factor for intestinal selection of preexisting endogenous *S. marcescens* resistant to tobramycin, with subsequent colonization of the respiratory tract.

The overall incidence of *S. marcescens* was 2.7 cases per 1000 patient days over an 8-year period. Comparatively, a Dutch cardiothoracic ICU reported an incidence of 6.7 cases per 1000 patient days in 2005, considering patients with samples taken >12 h post-admission and until 96 h post-discharge [14]. Another study spanning 2000–2005 across three adult acute care hospitals, a pediatric hospital, and community sites recorded a yearly incidence of 0.11 *Serratia* isolates per 1000 residents, with 65% being community-onset cases, but without notable seasonal or annual variations [15].

We suspected an aquatic reservoir to be linked to the 2017 outbreak, considering environmental sampling results, lapses in water-related care practices, the lack of other identified reservoirs, and the available literature. Indeed, a recent cartography of microbial ecology in hospitals observed *S. marcescens* in biofilms, often present in sinks and aerators [16]. Another review examining wastewater drain-related outbreaks from 1990 to 2018 identified an uncontrolled outbreak involving *Serratia* spp. despite sink cleaning and disinfection [17]. A similar review considering all Center for Diseases Control (CDC) investigations covering waterborne healthcare-associated infections from 2014 to 2017 included 134 investigations involving 1380 patients, with *Serratia* spp. implicated in 6 clusters affecting 49 patients [18].

Our study indicates that both behavioral and architectural interventions to mitigate a suspected aquatic reservoir did not effectively eradicate *S. marcescens*. In contrast, an outbreak involving 27 ICU patients with OXA-48 producing *S. marcescens* linked to sinks resolved with trap replacements, strict daily cleaning protocol with 1000 ppm hypochlorite, monthly sink trap surveillance, and educational efforts [19]. Many ICUs have transitioned to waterless environments, successfully reducing certain waterborne pathogens such as *Pseudomonas aeruginosa* [10,20], with an impact on MDR-GNB incidence [21,22]. In one of those studies, a decreasing number of *S. marcescens* infections was noted (from 20 to 13 cases) post-intervention [22].

Despite known aquatic reservoirs for *S. marcescens*, alternative unrecognized reservoirs and transmission routes may reduce the potential effectiveness of waterless care strategies. The epidemic curve and polyclonal strain distribution strongly suggest either a large, undetected reservoir of endogenous gastrointestinal carriage already present upon admission or an environmental source. Aside from the aquatic reservoir, neither extensive environmental sampling nor statistical analyses identified any other clear transmission pathway.

Regarding antibiotic selection pressure, our analysis observed a borderline protective effect of antibiotic exposure on *S. marcescens* incidence. Though difficult to interpret, this effect could be explained by the multi-susceptible profile of the strains retrieved in the ICU. Notably, *S. marcescens* incidence was also associated with the monthly use of SOD (containing colistin and tobramycin) in patients receiving more than 48 h of invasive ventilation. Effectively, *S. marcescens* is intrinsically resistant to colistin and can acquire resistance to tobramycin. The present study revealed an association between prescription of SOD and incidence of *S. marcescens*, which may also reflect an indirect association with prolonged ventilation. However, this association was not confirmed by the sub-cohorts, possibly reflecting a lack of power due to the relatively small sample size. A 21-year study examining the long-term effects of selective digestive decontamination showed limited ecological impact on potential pathogens and related selection of resistance, including resistance to tobramycin, gentamycin, polymyxin B, and colistin. Notably, *Serratia* spp. was excluded from this study due to its intrinsic resistance [23]. Another 5-year case-control study across 11 German ICUs assessed the impact of SOD (colistin, gentamycin, nystatin or amphotericin B) on 5034 mechanically ventilated patients, with 1694 not receiving SOD [24]. The incidence of *S. marcescens* was similar in patients with and without SOD, with 0.12 cases per 1000 patient days in both groups. In that study, non-statistically significant differences were obtained when considering patients hospitalized for more than 48 h before ICU admission.

Furthermore, the role of both invasive and non-invasive ventilation and the associated use of passive and active humidifiers in facilitating *S. marcescens* colonization of ventilatory circuits cannot be ruled out [25]. Acquisition via ventilatory circuits may occur through exogenous sources—primarily related to circuit maintenance—or endogenous mechanisms, such as aspiration of oropharyngeal secretions during nebulizer use. Exogenous acquisition is mitigated by hand hygiene, minimizing circuit manipulations, using a closed tracheal suction system (CTSS), and placing antibacterial filters at the inspiratory and expiratory circuit junctions. Endogenous pathways are addressed through bundled interventions including SOD, oral care, head-of-bed elevation (>30°), subglottic aspiration, appropriate cuff pressure, sedation monitoring, assessment for weaning, and early mobilization. These interventions have been previously implemented in our ICU through a successful bundle to prevent ventilator-associated pneumonia [12] Nevertheless, inconsistencies observed in using or changing heat and moisture exchanger (HME) filters and antibacterial filters, soiled heated humidifiers, and frequent circuit disconnections during the weaning phase may contribute to this colonization, although efforts have been made to optimize practices and reduce contamination. Unfortunately, we were not able to conduct a systematic assessment of such lapses, or microbiological sampling among colonized patients and ventilatory circuits.

We acknowledge further potential study limitations. First, potential unrecognized transmission pathways described above could have explained this high endemicity, highlighting the difficulty of controlling the spread of a multi-susceptible *S. marcescens* in a critical care environment. Second, detection bias may have artificially inflated the incidence rates, due to intensive sampling in our ICU. Third, the absence of systematic rectal screening raises the possibility of misclassification bias. Fourth, we missed some epidemiological and microbiological characteristics due to the retrospective nature of this study. Lastly, the study’s timeline coincided with the COVID-19 pandemic, which could have introduced additional bias by potentially impacting the effectiveness of waterless patient care, given the temporary increase in staff workload and the number of ventilated patients. Effectively, we observed from October 2020 a higher number of days spent on ventilation, higher SAPS II score, and a mean length of stay extended by at least 2 days (Table 1). However, statistical adjustment for this period was impossible due to the complete correlation of the pandemic period with the timing of our interventions (no COVID-19 cases occurred during the control period).

## 5. Conclusions

Behavioral interventions and transition to a waterless environment, aimed at addressing a presumed aquatic reservoir, were ineffective in curbing the endemic occurrence of polyclonal, multi-susceptible *S. marcescens* in our ICU. Undetected alternative routes of acquisition may have altered the effectiveness of waterless strategies, including invasive ventilation and ventilation-related practices such as SOD or the maintenance of ventilatory circuits.

## Figures and Tables

**Table 1:** Patient characteristics, infection control metrics, and staffing workload throughout the different study periods.

	Time Period 1—Baseline Period 1 January 2014–28 February 2018	Time Period 2—Behavioral Intervention 1 March 2018–30 September 2020	*p*-Value (Time Period 1 vs. 2)	Time Period 3—Architectural Intervention 1 October 2020–31 December 2022	*p*-Value (Time Period 1 vs. 3)	*p*-Value (Time Period 2 vs. 3)
Cases (*n*)	79	47		41		
Patient days (*n*)	30,588.96	20,613.07	0.02	15,031.4	0.18	0.002
*Serratia marcescens* incidence per 1000 patient days (*n*, IQR)	2.74 [0.00–3.65]	1.69 [1.17–4.08]	0.73	2.95 [0.00–4.50]	0.58	0.45
Ventilation days (median, IQR)	291.5 [246–333.5]	265 [219–295]	0.16	439 [360.5–488.5]	<0.001	<0.001
PRN score (mean, sd)	1161.42 [1137.94–1186.91]	1358.7 [1239.92–1425.65]	<0.001	1595.71 [1552.42–1645.51]	<0.001	<0.001
SAPS II (mean, sd)	40.88 [39.24–42.14]	45.53 [43.27–46.89]	<0.001	50.47 [48.7–51.94]	<0.001	<0.001
Length of stay in days (mean, sd)	3.75 [3.53–4.22]	3.8 [3.27–4.28]	0.49	5.72 [5.01–6.47]	<0.001	<0.001
Age (mean, sd)	60.19 [59.01–61.79]	60.44 [59.33–61.38]	0.86	60.4 [58.82–61.42]	0.87	0.75
Hand hygiene compliance (median, IQR)	64.55 [61.09–69.47]	62.29 [56.52–65.71]	0.05	68.33 [57.91–73.99]	0.69	0.15
Antibiotic consumption (DDD per 1000 patient days) (median, IQR)	144.86 [115.46–170.06]	110.71 [98.77–123.96]	<0.001	128.42 [103.84–149.23]	0.14	0.03
Number of SOD prescriptions (median, IQR)	60.5 [49–76]	64 [58.5–75.5]	0.19	108 [84–122.5]	<0.001	<0.001

PRN: project research of nursing; SOD: selective oral decontamination; SAPS II: simplified acute physiology score II; DDD: defined daily dose; IQR: interquartile range.

## Data Availability

The data presented in this study are available on reasonable request from the corresponding author.

## Supplementary Materials

The following supporting information can be downloaded at: https://www.mdpi.com/article/10.3390/pathogens14040363/s1. References [26,27,28,29] are cited in the Supplementary Materials.
