# Supplementary material for: Impact of Multifaceted Interventions Including Waterless Patient Care on Endemic Occurrence of Serratia marcescens in an Intensive Care Unit
Source: Pathogens. 2025 Apr 8;14(4):363. doi: 10.3390/pathogens14040363 (PMC12030487; doi:10.3390/pathogens14040363)
Supplement: Supplementary file 1 [file pathogens-14-00363-s001.zip › pathogens-3553963-supplementary-final.pdf]

Supplementary Online Content

Impact of Multifaceted Interventions Including Waterless Patient Care on Endemic Occurrence of *Serratia marcescens* in an Intensive Care Unit

R. Martischang [1], G. Catho [1,2], A. Cherkaoui [3], F. Boroli [4], N. Buetti [1], J. Pugin [4] and S. Harbarth [1]

1. Infection Control Program, Geneva University Hospitals and Faculty of Medicine, WHO Collaborating Center, Geneva, Switzerland

2. Infectious Diseases Division, Central Institute, Valais Hospital, Sion, Valais

3. Bacteriology Laboratory, Geneva University Hospitals and Faculty of Medicine, Geneva, Switzerland

4. Division of Intensive Care Medicine, Geneva University Hospitals and Faculty of Medicine, Geneva, Switzerland

Supplementary file S1. Antibiotic agents at risk for *S. marcescens* selection

The following antibiotic compounds were considered at-risk to select intrinsically resistant *S.marcescens :* colistin, polymyxin E, macrolides (azithromycin, clarithromycin, erythromycin), nitrofurantoin, sulfamethoxazole-trimethoprim, penicillins (amoxicillin, co-amoxicillin), and 1st and 2nd generation cephalosporins (cefazolin, cefuroxime) [26]. In addition, due to high resistance levels observed in Switzerland (only 58% were susceptible, with a MIC90 at 4 mg/l) [27], we also included the aminoglycosides tobramycin and amikacin, which are known to select acquired resistance, mostly through a chromosomal and/or plasmidic AAC(6')-lb enzyme.

Supplementary file S2. Environmental investigations

<u>1st environmental screening survey: 18.08.2017</u>

This screening included 56 samples from sinks (n=10, basin, faucet, drain, P-traps), surfaces (n=16), high-touch surfaces (n=8; e.g. computer keyboards, stethoscopes), and solutions (n=22; soap, disinfecting solutions, echography gel). Ventilators were not sampled, since multiple machines were used for affected patients.

<u>2nd environmental screening survey: 01.08.2018</u>

This screening included 30 samples: 17 from five sinks (faucet, drain, basin, P-trap) and 13 from high-touch surfaces (keyboards, mice, fingerprint detectors, fan screen, monitor screen), oxygen equipment (connector, mask), and toiletry/oral care items from patient rooms. Only two sink samples tested positive in the second screening survey.

<u>Post-interventional screening :</u>

Due to the low yield of both screening surveys, a post-intervention screening was not performed.

<u>Microbiological analyses</u>

Sample collection was performed in 2017 using ESwabs® (COPAN) using a predefined protocol [28], and in 2018 using flocked nylon swabs (QUANTISWAB IRR®, Biomérieux). The samples were cultured in 2017 on non-selective agar plates, with the addition in 2018 of selective media specific for *S. marcescens* (CHROMID CPS ELITE OPAQUE®, Biomérieux and HEKTOEN ENTERIC AGAR®, BIO-RAD). Subsequent identification of any suspicious colonies was performed via MALDI-TOF analysis.

Supplementary file S3. Genomic methods

To explore potential institution-wide transmission during the initial 2017 outbreak, we assessed genetic relatedness among 70 *S. marcescens* strains collected at HUG from 2015 to 2018, which included 13 clinical strains from ICU patients and 3 environmental ICU strains. For genomic sequencing, selected isolates were processed using the iGE3/CMU genomic platform on an Illumina MiSeq system (TruSeq DNA Nano, 2 x 300). The FASTQ reads, retrieved from the iGE3 servor, underwent filtering and trimming (Trimmomatic v 0.36) and were then assembled into contigs (SPAdes genome assembler v3.11.0). Similarity analysis (Average Nucleotide Identity), cgMLST (core genome multilocus sequence typing) and Minimum Spanning Tree were then performed. The reference for the cgMLST was a previously sequenced strain SM-39, analyzed using SeqSphere (Ridom SeqSphere+ version 4.1.9). A total of 70 strains and 1435 loci were included in the cgMLST analysis.

Supplementary file S4. Statistical analysis – Selection of the best model (R code)

```
predictors<-c("SAPSII_moyen", "month", "PRN_moyen", "LOS_moyen", "compliance", "nventilationday",
"DDD_rate", "n_SOD", "TimePeriod*nmonth", "month")
combinations_list <- list()
for (i in 1:length(predictors)) {
combinations_list[[i]] <- combn(predictors, i, function(x) unique(c("TimePeriod", x)), simplify = FALSE)}
combinations <- unlist(combinations_list, recursive = FALSE)
models <- lapply(combinations, function(x) {
glm(as.formula(paste("n ~ offset(log(journee)) +", paste(x, collapse = " + "))),
family = poisson, data = smarincpermonth)})
aic_values <- lapply(models, AIC)
best_indices <- order(unlist(aic_values))[1:5]
```

```
best_models <- models[best_indices]

best_model_ci <- lapply(best_models, function(model) {list(round(ci.lin(model, Exp=T), 3),

AIC=AIC(model))})
```

Table S1. Key interventions and investigations to mitigate the spread of *S. marcescens*

**Existing protocols**		
**Ventilation circuit management and VAP prevention bundle**		Bundled interventions were performed, including hand hygiene, minimizing circuit manipulations, using closed tracheal suction system (CTSS), and placing antibacterial filters at the inspiratory and expiratory circuit junctions, selective oral decontamination, oral care, head-of-bed elevation (>30°), subglottic aspiration, appropriate cuff pressure, sedation monitoring, assessment for weaning, and early mobilization [12].
**Hand hygiene protocols**		Hand hygiene was performed according to the World Health Organization "My 5 moments for hand hygiene" concept. The adherence was assessed prospectively by specialized nurses [29].
**Key interventions**		
**Start**	**End**	**Detail**
01.11.2017	ongoing	Educational rounds to reinforce training of nursing staff regarding standard precautions, care management, standard and transmission-based precautions
01.02.2018	ongoing	Educational rounds to reinforce compliance with hand hygiene, proper use of gloves and aseptic care procedures while using water.
01.03.2018	ongoing	Implementation of procedures regarding water-related practices
28.03.2020	ongoing	Separation of non-contaminated and contaminated areas and tasks, dedicated storage space > 1 m from sinks, splash gard
18.09.2020	09.10.2020	Environmental disinfection
18.09.2020	22.09.2020	Limited ICU bed closures
21.09.2020	28.09.2020	Sink removal from patient rooms, limitation of the use of remaining sinks for hand hygiene when specifically indicated only, procedures and teaching for waterless patient bathing

ICU: intensive care unit. VAP : ventilation associated pneumonia.

Table S2: Characteristics of the cohorts

	**Main cohort**	**SCohort1**	**SCohort 2**
Sampling date	January 2014 to December 2022		
Definition	Positive screening or clinical culture positive for *S.marcescens* sampled >48 hours after ICU admission, up to 14 days after ICU discharge	Positive screening or clinical culture positive for *S.marcescens* sampled from admission up to 14 days after discharge	Previously negative patients[a] with a positive screening or clinical culture for *S.marcescens* sampled >72 hours after ICU admission up to ICU discharge
Cases (n)	167	233	46
Incidence of *S. marcescens* per 1'000 patient days (median, IQR)	2.67 [0.00-3.98]	3.17 [1.57-5.24]	0.00 [0.00-1.57]
Median delay from admission to sampling (n, IQR)	8.0 [3.9-13.3]	4.7 [1.8-10.8]	10.2 [7.8-19.3]
Median delay (in days) from negative sample to positive sample	NA	NA	6.8 [4.3-13.7]
Sampling site distribution	Respiratory: 70.7% (118/167)<br>Blood: 13.2% (22/167)<br>Urine: 7.8% (13/167)<br>Undetermined: 8.4% (14/167)	Respiratory: 77.7% (181/233)<br>Blood: 9.9% (23/233)<br>Urine: 6.0% (14/233)<br>Undetermined: 6.4% (14/233)	Respiratory: 96% (44/46)<br>Urine: 4% (2/46) [a]

[a] Considering a negative sample from a respiratory or urine site, from the same subsequent colonization or infection site.

Table S3. Multivariate Poisson regression model of *Serratia marcescens* incidence in the ICU

	Incidence Ratios [95%CI]	p-value
Behavioral intervention	1.02 [95%CI 0.33-3.11]	0.97
Architectural intervention	4.25 [95%CI 0.59-30.56]	0.15
PRN score	1.00 [95%CI 0.99-1.00]	0.25
Hand hygiene compliance	1.01 [95%CI 0.97-1.05]	0.70
Antibiotic consumption (DDD per 1'000 patient days)	0.99 [95%CI 0.97-1.00]	0.052
Number of SOD prescription	1.01 [95%CI 0.98-1.03]	0.58

PRN: project research of nursing; SOD: selective oral decontamination; DDD: defined daily dose.

Table S4. Multivariate Poisson regression model of *S.marcescens* incidence in SCohort1

	**Incidence Ratios [95%CI]**	**p-value**
Behavioral intervention	1.01 [95%CI 0.61-1.69]	0.95
Architectural intervention	1.15 [95%CI 0.56-2.38]	0.70
PRN score	1.00 [95%CI 1.00-1.00]	0.06
Hand hygiene adherence	1.01 [95%CI 1.00-1.03]	0.41
Antibiotic consumption (DDD per 1'000 patient days)	0.99 [95%CI 1.00-1.00]	0.80
Number of SOD prescriptions[a]	1.01 [95%CI 1.00-1.03]	0.01
January	2.36 [95%CI 1.01-5.48]	0.046
February	0.30 [95%CI 0.09-1.00]	0.05
March	0.93 [95%CI 0.37-2.32]	0.88
April	0.94 [95%CI 0.35-2.51]	0.90
May	1.31 [95%CI 0.54-3.17]	0.55
June	0.99 [95%CI 0.39-2.55]	1.00
July	1.84 [95%CI 0.79-4.26]	0.16
August	*reference*	
September	1.05 [95%CI 0.41-2.68]	0.91
October	1.50 [95%CI 0.64-3.50]	0.34
November	0.72 [95%CI 0.28-1.86]	0.50
December	2.15 [95%CI 0.92-5.00]	0.08

PRN: project research of nursing; SOD: selective oral decontamination; DDD: defined daily dose.

[a] Per prescription increment.

Table S5. Multivariate Poisson regression model of *S.marcescens* incidence in SCohort2

	Incidence Ratios [95%CI]	p-value
Behavioral intervention	0.57 [95%CI 0.15-2.20]	0.41
Architectural intervention	2.93 [95%CI 0.31-27.29]	0.34
PRN score	1.00 [95%CI 0.99-1.01]	0.88
Hand hygiene adherence	1.00 [95%CI 0.96-1.04]	0.97
Ventilation days	0.99 [95%CI 0.98-1.00]	0.11
Antibiotic consumption (DDD per 1′000 patient days)	0.99 [95%CI 0.97-1.00]	0.08
Number of SOD prescription [a]	1.02 [95%CI 0.99-1.06]	1.02

PRN: project research of nursing; SOD: selective oral decontamination; DDD: defined daily dose.

[a] Per prescription increment

Figure S1: cgMLST analysis of a convenience sample of *S.marcescens* strains isolated at Geneva University Hospitals from 2015 to 2018, incorporating strains collected in the ICU.

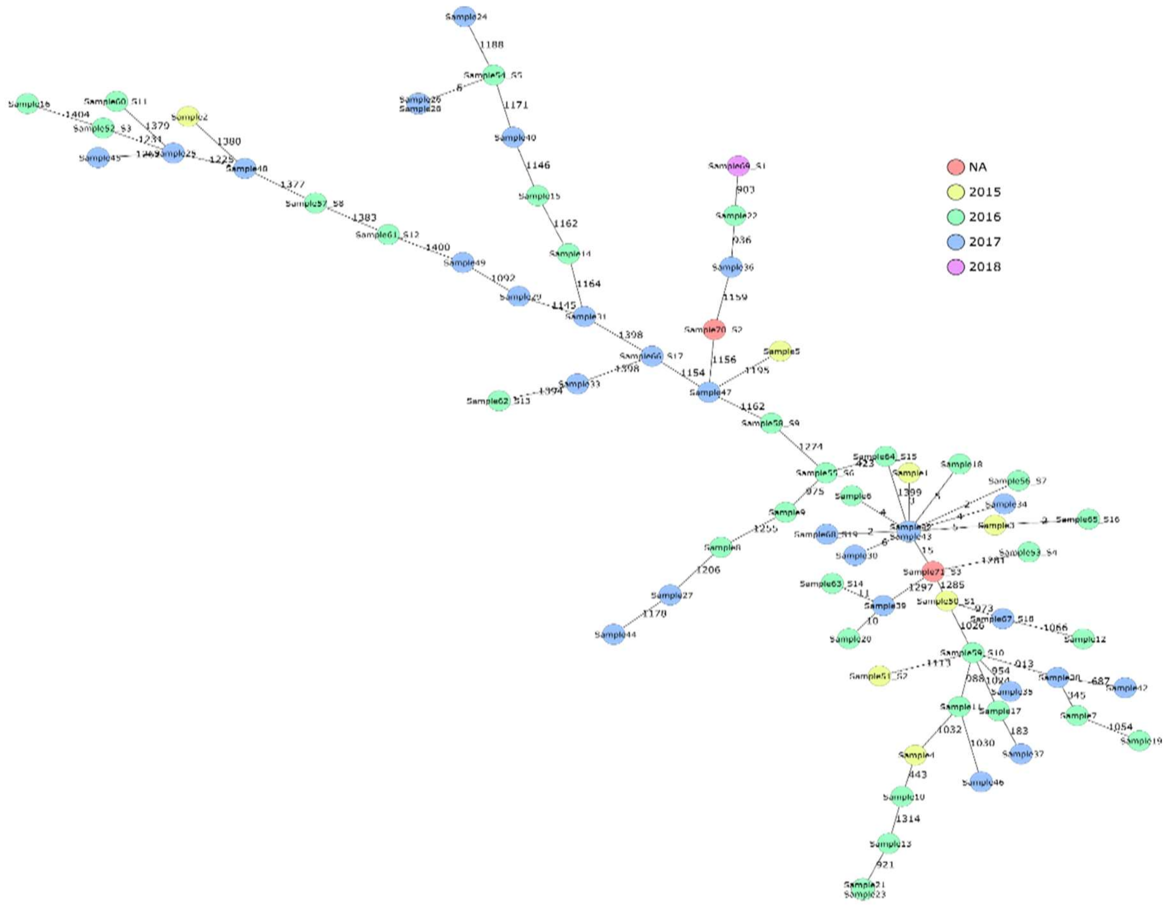

Projects: S. marcescens all samples (Serratia marcescens)
Comparison Table Retrieval: S. marcescens all samples [unstored]
Task Templates: S. marcescens cgMLST 3233 targets SM39
Comparison Table created: Nov 12, 2018 2:24 PM (v4.1.9_(2017-10))
Ridom SeqSphere+ MST for 70 Samples based on 1435 columns, no missing values
Distance based on columns from S. marcescens cgMLST 3233 targets SM39 (1435)
For citing correctly in publications the tools used for this analysis see menu Help | Citations.
Color grouped by column "Collection Date":

From 2015 through 2018, we identified 11 genetically similar *S.marcescens* strains (≤15 SNP differences) across different patients and departments, including 6 clinical strains retrieved from separate areas of the ICU. Among the three environmental *S.marcescens* strains analyzed (16-21 SNP differences from clinical strains), on was part of an ICU cluster.
